# Changes in EEG Activity Following Live *Z*-Score Training Predict Changes in Persistent Post-concussive Symptoms: An Exploratory Analysis

**DOI:** 10.3389/fneur.2022.714913

**Published:** 2022-03-21

**Authors:** Jamie N. Hershaw, Candace A. Hill-Pearson

**Affiliations:** ^1^Defense Health Agency (DHA) Traumatic Brain Injury Center of Excellence, Fort Carson, CO, United States; ^2^General Dynamics Information Technology, Falls Church, VA, United States

**Keywords:** traumatic brain injury, post-concussive symptoms, neurofeedback, EEG, lasso regression

## Abstract

A specific variant of neurofeedback therapy (NFT), Live *Z*-Score Training (LZT), can be configured to not target specific EEG frequencies, networks, or regions of the brain, thereby permitting implicit and flexible modulation of EEG activity. In this exploratory analysis, the relationship between post-LZT changes in EEG activity and self-reported symptom reduction is evaluated in a sample of patients with persistent post-concussive symptoms (PPCS). Penalized regressions were used to identify EEG metrics associated with changes in physical, cognitive, and affective symptoms; the predictive capacity of EEG variables selected by the penalized regressions were subsequently validated using linear regression models. Post-treatment changes in theta/alpha ratio predicted reduction in pain intensity and cognitive symptoms and changes in beta-related power metrics predicted improvements in affective symptoms. No EEG changes were associated with changes in a majority of physical symptoms. These data highlight the potential for NFT to target specific EEG patterns to provide greater treatment precision for PPCS patients. This exploratory analysis is intended to promote the refinement of NFT treatment protocols to improve outcomes for patients with PPCS.

## Introduction

A ubiquitous pathophysiological consequence of mild traumatic brain injury (mTBI) is dysfunctional neural activity (1–3). For many individuals who sustain mTBI, this neural dysfunction and its associated cognitive, physical, and behavioral symptoms are transient and resolve within a matter of weeks or months. However, a minority of patients–5–30%—experience persistent post-concussive symptoms (PPCS) beyond the typical recovery period (4–6) and these symptoms are often associated with residual neural dysfunction (7–10).

Research efforts are underway to identify and validate effective treatments to improve a range of neurobehavioral symptoms in patients with PPCS. One promising treatment is neurofeedback therapy (NFT), with several studies reporting that NFT reduces symptoms in patients with TBI (11–17). NFT is a non-pharmacological treatment that uses operant conditioning to train patients to autonomously modulate neural activity (18, 19). The general premise of NFT is that patients receive positive feedback when their EEG activity achieves a desired pattern; through this process, patients implicitly learn to regulate their EEG activity to achieve desired behavioral goals. There are numerous variations of NFT that target different aspects of neural activity, including specific oscillatory frequencies (20, 21) and/or specific networks or regions of the brain (22, 23).

One type of NFT, Live *Z*-Score Training (LZT), is unique from common variants of NFT for its real-time, continuous calculation of *Z*-scores of EEG activity (24, 25). *Z*-scores are calculated for six EEG metrics in ten frequency bands over all channels included in the EEG montage; in a 19-channel montage, LZT produces 5,700 Z-scores (Table 1). Participants receive positive feedback when a pre-specified percentage (e.g., 60%) of all computed *Z*-scores falls within a pre-specified range (e.g., ±1 SD) of the normative mean (24, 25). Training can be configured to either target specific EEG activity or remain agnostic to what EEG metrics are targeted. With this latter approach, patients are trained to normalize activity in some but not all frequency bands and/or brain regions, as normalization of only a subset of EEG metrics is required for reinforcement. Because any combination of *Z*-scores can satisfy the criteria to receive positive feedback, there can be substantial variability between patients in the strategies they implicitly and flexibly choose for normalizing neural activity (24, 25). For example, a patient who normalizes brain-wide alpha activity and a patient who normalizes theta and beta activity over central and parietal regions may be equally successful at completing LZT. However, it is unknown how various strategies for EEG normalization, when not specified and directly targeted by NFT, correspond to symptom improvements.

**Table 1:** Live *Z*-score training EEG measurement parameters.

**Frequency bands**	**Metrics**	**Channels**
Delta (1–3 Hz)	Absolute power	FP1
Theta (4–7 Hz)	Relative power	F3
Alpha (8–12 Hz)	Power ratios	C3
Alpha-1 (8–10 Hz)	Asymmetry^*^	P3
Alpha-2 (10–12 Hz)	Phase delay^*^	O1
Beta (12–25 Hz)	Coherence^*^	F7
Beta-1 (12–15 Hz)		Fz
Beta-2 (15–18 Hz)		T3
Beta-3 (18–25 Hz)		T5
High Beta (15–30 Hz)		FP2
		F4
		C4
		P4
		O2
		F8
		Cz
		Pz
		T4
		T6

* *Metrics computed for each possible electrode pair*.

Consistent with previous studies of NFT, a feasibility analysis recently conducted in our laboratory demonstrated that LZT was associated with symptom reduction in a sample of active duty service members and veterans with PPCS (26). Here we report results of an exploratory analysis of data from the feasibility study to (1) characterize changes in resting state EEG activity in patients with PPCS following completion of LZT and (2) evaluate how changes in EEG activity are associated with changes in symptomatology. Results from this analysis may be helpful to identify EEG activity that can be targeted by NFT to customize symptom-specific treatment in PPCS patients.

## Methods

### Design

This study was approved by the Madigan Army Medical Center's Institutional Review Board. Here we provide a summary of study methods; full details are provided in Hershaw et al. (26). This study used a single-group design wherein participants received LZT. Participants completed pre-treatment (T1), post-treatment (T2) and follow-up (T3) evaluations that included self-report symptom questionnaires, a full neuropsychological battery, a physiological stress test, and resting state EEG recording. T1 evaluations were conducted no more than 4 weeks prior to the start of LZT; T2 evaluations were conducted between 1 and 4 weeks following treatment; and T3 evaluations were conducted between 11 and 15 weeks following treatment.

### Participants

Participants were recruited from Fort Carson Army Post and the surrounding Colorado Springs, CO area using posted recruitment material and provider referrals. To be eligible for study participation, individuals had to have active duty or veteran status, be 18–50 years old, have a history of mild to moderate TBI as defined by the American Congress of Rehabilitation Medicine (27) 3 months to 5 years prior to enrollment, and currently endorse post-concussive symptoms to include emotion dysregulation. Individuals with unstable medical or psychiatric conditions, who failed symptom validity tests, endorsed alcohol or substance abuse, or used medications known to interfere with EEG recordings were excluded from participation. A total of 38 individuals met eligibility requirements and agreed to undergo LZT.

### LZT Protocol

LZT training stimuli were delivered via a video chosen by participants from the BrainMaster BrainAvatar software system (Bedford, OH: BrainMaster Technologies). As the video played, EEG data were recorded using the BrainMaster Discovery 24E amplifier using a 19-channel montage adhering to the 10–20 international electrode placement system. EEG data were decomposed into 10 frequency bands to obtain measures of absolute power, relative power, and power ratios at each electrode, and asymmetry, phase delay, and coherence for every electrode pair (Table 1). Given every possible combination of metrics and electrode pairs, 5,700 EEG metrics were computed and compared continuously in real-time to a normative database [Applied NeuroScience BrainMaster *Z*-Score Dynamic Link Library (Largo, FL: Applied Neuroscience, Inc.)] to derive *Z*-scores. When participants' EEG activity approximated normative values (as defined by pre-specified criteria described below), they received positive feedback in the form of visual or audio cues in the video.

To receive positive feedback, the following three criteria must have been met: (1) *Z*-scores had to fall within a “target window” of ±0.9 standard deviations of the normative mean; (2) based on a titrated threshold, a certain percentage of the 5,700 *Z*-scores had to fall within the target window 40–60% of time; and (3) a variable percentage of *Z*-scores that were outside of the target window (“outliers”) had to move toward the target window. The required percentage of outlier *Z*-scores moving toward the target window was adjusted continuously, as needed, such that participants received 10–15 reinforcements per minute. The target window was held constant throughout treatment, but the threshold for percentage of *Z*-scores needing to fall within that window varied depending on performance. The duration of each treatment session increased from 10 min at the first session to 30 min by session 6 or 7. LZT treatment took place over a period of 6 weeks. Treatment completion was defined as completing at least 15 treatment sessions; however, participants were encouraged to complete as many sessions as possible, up to 20.

### Self-Reported Symptom Questionnaires

Given that many functional domains can be affected in patients with PPCS, self-report symptom questionnaires were selected to assess a broad range of symptoms. The following symptoms were assessed: (1) post-traumatic stress symptoms [PTSD Checklist-Military version, PCL-M; (28)]; (2) depressive symptoms [Patient Health Questionnaire-9, PHQ-9; (29)]; (3) neurobehavioral symptoms [Neurobehavioral Symptom Inventory, NSI; (30)]; (4) sleep quality [Medical Outcomes Study Sleep Scale, MOS-Sleep; (31)]; (5) pain [Chronic Pain Grade questionnaire, CPG; (32)]; and (6) migraines [Migraine Disability Assessment Scale, MIDAS; (33)]. The following symptom scores were derived from these questionnaires in accordance with their respective scoring manuals to be included in analyses: PCL-M total; PHQ-9 total; NSI somatic subscale; NSI affective subscale; NSI cognitive subscale; NSI vestibular subscale; MOS-Sleep total; CPG pain intensity subscale; CPG pain-related disability subscale; and MIDAS total. A decrease in all scores represents symptom improvements.

### Resting State EEG Data Acquisition and Pre-processing

As part of the pre- and post-treatment evaluations, eyes open and eyes closed resting state EEG data were recorded. Data were recorded at a 1,000 Hz sampling rate using NeuroScan acquisition system (Victoria, Australia: Compumedics Neuroscan) with a 64-channel montage adhering to the 10–20 international electrode placement system. Data were processed offline using EEGlab (34). Data were downsampled to 256 Hz and a 1–100 Hz bandpass filter was applied. Data were then cleaned using the EEGlab clean_rawdata function (35), referenced to the common average, and submitted to an independent components analysis to identify and remove horizontal and vertical ocular artifacts from the data. Cleaned data were then submitted to the bandpower function in MATLAB (Version 2014b, Natick, MA: Mathworks), whereby spectral power density is estimated using a periodogram method.

### EEG Variable Quantification

In an effort to reduce the computational burden and the risk of committing Type 1 errors in this exploratory analysis, we analyzed eyes open resting state data; the eyes open condition was chosen due to its properties of having fewer ocular artifacts (36), reduced large-amplitude alpha rhythm that is dominant during eyes closed resting periods (36), and greater sensitivity to neural dysfunction compared to eyes closed EEG activity (37). Resting state EEG data was quantified to maximize fidelity to the EEG metrics for which *Z*-scores were computed as part of LZT training. The system used to record resting state EEG data had a 64-channel montage; the system used to deliver LZT had a 19-channel montage (see Table 1). We limited our quantification of resting state EEG data to those channels in the 64-channel system that were also represented in the 19-channel system; however, channels T3, T4, T5, and T6 were included in the 19-channel system but not the 64-channel system. As a substitute for these channels, we included channels T7 and T8 in our quantification, as these channels were included in the 64-channel system but not in the 19-channel system. This resulted in 17 channels included in the resting state EEG analysis.

As a further effort to reduce the computational burden and the risk of committing Type 1 errors in this exploratory analysis, we analyzed a subset of the 5,700 EEG variables that are included in LZT training. Resting state EEG data were decomposed into the same 10 frequency bands that were included in LZT training (see Table 1), as well as total power (1–30 Hz). We quantified absolute power, relative power, power ratios, and asymmetry metrics. Absolute power was measured for each of the 10 frequency bands and total power. Relative power was measured as the absolute power in each of the 10 frequency bands divided by total power. Five absolute power ratios were computed: theta/beta, theta/alpha, alpha/beta, delta/theta, and delta/alpha. The first four ratios were selected because they are the most commonly reported power ratios (38) and the latter ratio was selected in light of evidence that it is related to rehabilitation following TBI (39). All power metrics were quantified for each of the 17 channels, resulting in 442 power metrics. Additionally, we computed lateral asymmetry ratios for frontal (FP2–FP1), parietal (P4–P3), and temporal (T8–T7) regions for each of the 10 frequency bands and total power. These asymmetry-based metrics brought the total number of EEG variables to 475.

### Statistical Analysis

For the purpose of this analysis, we limited our comparison to pre- (T1) and post-treatment (T2) EEG metrics. EEG metrics obtained during follow-up (T3) were not included in the analysis because of existing evidence that changes in EEG activity following NFT are often not sustained and may even rebound, despite observing sustained clinical effects (40). This dissociation is attributed to a homeostatic mechanism that permits “re-normalization” of EEG activity while maintaining behavioral gains (41, 42); thus long-term measures of EEG activity may not validly represent the association between NFT-induced EEG alterations and symptom resolution.

To characterize post-treatment changes in EEG power, we compared EEG variables between T1 and T2 using paired samples *t*-tests. Because of the exploratory nature of this analysis, we report results both without a correction for multiple comparisons [see (43), for a thorough discussion on this topic] and with a Benjamini-Hochberg to correct for a False Discovery Rate (FDR) of 0.05 (44).

Change scores (T2–T1) were computed for all EEG variables (*n* = 475) and all symptom scores (*n* = 10). To identify what, if any, changes in EEG activity predict changes in symptomatology, a separate lasso regression was conducted for each symptom change score with all EEG change scores entered as predictor variables. Lasso regressions are optimal for variable selection when there is a large number of predictor variables (e.g., more than the number of observations) and high levels of multicollinearity. Lasso regression models impose a penalty, λ, on the value of coefficients that do not contribute to the prediction of an outcome variable, thereby shrinking to zero the coefficients of all non-predictive variables. The optimal value of λ is that which minimizes the lasso function in Equation 1.1. Thus, any variable with a non-zero coefficient is a significant predictor of the outcome. The result is a parsimonious model that includes only unrelated variables that significantly contribute to the prediction of the outcome. In effect, it is a solution to the problem of overfitting when there are a large number of predictors entered into a model.


(1) $$\begin{array}{l} \overset{n}{\underset{i=1}{\sum }}{\left(Y_{i}-\underset{j}{\sum }X_{ij}\beta_{j}\right)}^{2}+\lambda \overset{p}{\underset{j=1}{\sum }}|\beta_{j}| \end{array}$$

The degree of coefficient shrinkage increases as λ, the penalty parameter, increases for a given value of α (here, α = 1). To determine the value of λ that would produce the optimal degree of shrinkage, we used 10-fold cross-validation for each of the lasso regressions, wherein values of λ were tested on 10 random subsets of the dataset to identify the value that yielded the smallest mean square error (MSE) of prediction. The predictor coefficients that are non-zero at this optimal value of λ reflect the EEG variables selected by the model as significant predictors of the outcome. Following each lasso regression, EEG variables that were selected by the regressions were then submitted to a multiple linear regression on the symptom change score using the full dataset to further validate their predictive capacity. All regression models were tested and are reported using standardized (*z*-score) variables.

## Results

Twenty-seven participants completed LZT treatment. One participant was excluded from analyses for unusable resting state EEG data. The final sample included in this exploratory analysis consisted of 22 males and four females with a mean age of 35.54 years (SD = 7.20). The mean time since injury to study enrollment ranged from 3 to 45 months (M = 14.16, SD = 12.82).

Of the 475 EEG variables tested, 31 (6.5%) changed significantly between T1 and T2 (Table 2); when a correction for FDR was applied, 27 (5.7%) of these variables remained significant. In the frontal and temporal regions, significant differences were noted almost exclusively for alpha metrics; in contrast, changes in additional frequency bands were observed throughout central and parietal regions.

**Table 2:** EEG variables with significant pre-post treatment difference.

**Region**	**Channel**	**Frequency**	***t*-statistic**	***p*-value**	**B-H**
Frontal	FP1	Alpha (rel)	3.27	0.003	0.003
		Alpha-1 (rel)	3.5	0.002	0.002
		Alpha-2 (rel)	2.19	0.038	0.040
		Alpha/Beta ratio	2.49	0.020	**0.023**
	FP2	Alpha (rel)	2.75	0.011	0.011
		Alpha-1 (rel)	2.95	0.007	0.008
	F3	Alpha-1 (rel)	2.21	0.036	0.037
	Fz	Delta (rel)	−2.45	0.022	0.027
		Alpha-1 (rel)	2.85	0.009	0.010
		Theta/Beta ratio	2.16	0.040	**0.044**
		Delta/Alpha ratio	−2.36	0.026	0.031
	F8	Alpha-1 (rel)	2.22	0.036	0.039
		Theta/Alpha ratio	−2.4	0.024	0.029
		Delta/Alpha ratio	−2.49	0.020	**0.024**
Central	C3	Alpha-1 (rel)	2.07	0.049	0.050
	Cz	Delta (rel)	−2.08	0.048	0.048
		Alpha (rel)	2.17	0.040	**0.045**
		Alpha-1 (rel)	2.76	0.011	0.013
	C4	Theta (rel)	2.48	0.020	0.026
		Alpha-1 (rel)	3.16	0.004	0.005
		Theta/Beta ratio	3.21	0.004	0.006
Parietal	P3	Alpha-1 (rel)	2.77	0.011	0.015
	Pz	Alpha-1 (rel)	2.18	0.039	0.042
	P4	Delta (rel)	−2.24	0.034	0.035
		Alpha-1 (rel)	2.53	0.018	0.021
		Theta/Beta ratio	2.55	0.017	0.019
		Delta/Theta ratio	−2.7	0.012	0.016
	Asymmetry (P4–P3)	Delta (abs)	−2.09	0.047	0.047
		Beta (abs)	−2.35	0.027	0.032
		High Beta (abs)	−2.31	0.029	0.034
Temporal	T8	Alpha-1 (rel)	2.61	0.015	0.018

*Df, 25; rel, relative power; abs, absolute power; negative t-value indicates metric decreased between T2 and T1; B-H, Benjamini-Hochberg critical value*.

*Bold text indicates non-significance after applying the Benjamini-Hochberg correction*.

**Figure 1:**
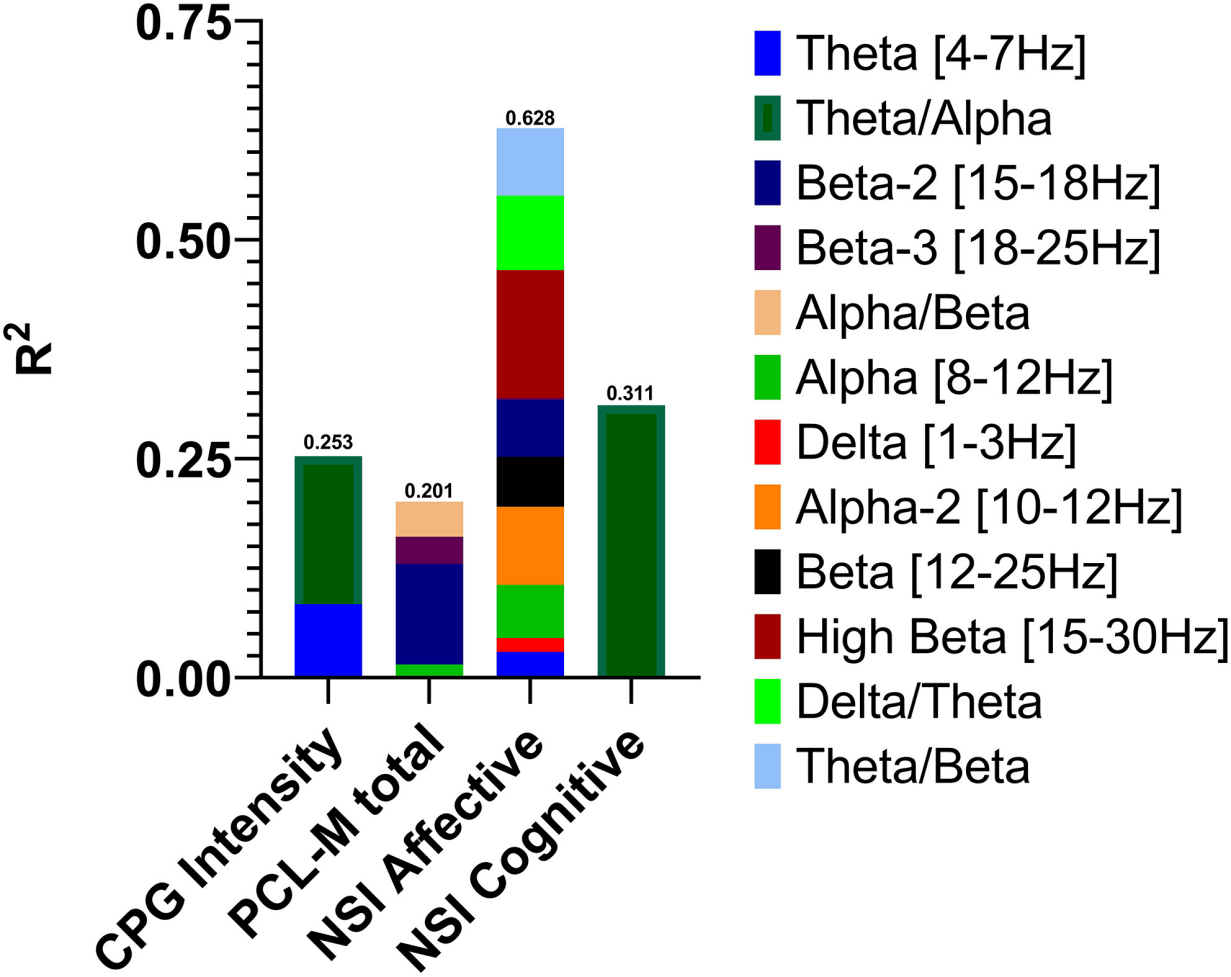
Variance in post-treatment changes in pain intensity, post-traumatic stress symptoms, affective symptoms, and cognitive symptoms accounted for by post-treatment changes in EEG activity in specific frequency bands and power ratios.

Descriptive statistics for symptom change scores are provided in Supplementary Table A. Improvements in pain intensity (Table 3) were predicted with a small margin of error by changes in lateral frontal and occipital theta/alpha ratio and occipital theta; 25% of the variance in pain reduction was accounted for by these EEG metrics. Outcomes related to emotion regulation, including PTSD-related symptoms (Table 4) and affective symptoms (Table 5), were predicted with high accuracy: nearly 20% of variance in changes in PTSD-related symptoms and almost 63% of the variance in changes in affective symptoms were explained by changes in several EEG metrics, predominantly beta-related metrics. Improvements in self-reported cognitive symptoms (Table 6) were predicted by theta/alpha ratio over the medial parietal region, with these metrics accounting for 31% of the variance in symptom reduction. Figure 1 depicts the amount of variance in each of these four outcomes that is accounted for by post-treatment changes in specific frequency bands and ratios. Descriptive statistics of change scores for all EEG variables that were selected as predictors by the lasso regressions are provided in Supplementary Table B.

**Table 3:** Regressions on CPG intensity.

**Elastic net regression**				**Linear regression**			
**λ**	**α**	**DF**	**MSE**	***R* ^2^**	***F***	***p***	**MSE**
0.343	1	4	1.078	0.253	1.776	0.171	0.890
**Selected predictors**		**Coefficient at** **λ**		**Predictors**		**Beta**	**95% CI**
Theta/Alpha ratio, F3		0.053		Theta/Alpha ratio, F3		0.320	−0.156 to 0.797
Theta/Alpha ratio, O1		0.097		Theta/Alpha ratio, O1		0.147	−0.346 to 0.640
Theta/Alpha ratio, F8		0.211		Theta/Alpha ratio, F8		−0.107	−0.579 to 0.364
Theta (rel), O1		0.030		Theta (rel), O1		0.287	−0.182 to 0.757
				(Constant)			−0.385 to 0.385

*rel, relative power; abs, absolute power*.

**Table 4:** Regressions on PCL-M.

**Elastic net regression**				**Linear regression**			
**λ**	**α**	**DF**	**MSE**	***R* ^2^**	***F***	***p***	**MSE**
0.351	1	4	0.920	0.201	1.373	0.277	0.944
**Selected predictors**		**Coefficient at λ**		**Predictors**		**Beta**	**95% CI**
Beta-3 (rel), F7		−0.1686		Beta-3 (rel), F7		−0.114	−0.555 to 0.327
Alpha/Beta ratio, P4		−0.0247		Alpha/Beta ratio, P4		0.148	−0.454 to 0.750
Alpha (rel), Cz		−0.0204		Alpha (rel), Cz		0.055	−0.530 to 0.639
Beta-2 (abs), T7		−0.1156		Beta-2 (abs), T7		0.423^*^	0.004–0.842
				(Constant)			−0.396 to 0.396

* *p < 0.05; rel, relative power; abs, absolute power*.

**Table 5:** Regressions on NSI affective.

**Elastic net regression**				**Linear regression**			
**λ**	**α**	**DF**	**MSE**	***R* ^2^**	***F***	***p***	**MSE**
0.150	1	12	0.799	0.628	1.830	0.147	0.715
**Selected predictors**		**Coefficient at λ**		**Predictors**		**Beta**	**95% CI**
Delta (rel), FP1		0.0866		Delta (rel), FP1		0.064	−0.366 to 0.494
Beta-2 (rel), F3		0.0834		Beta-2 (rel), F3		0.271	−0.301 to 0.843
Beta (rel), C3		0.1116		Beta (rel), C3		−0.236	−0.866 to 0.394
High Beta (rel), C3		0.0521		High Beta (rel), C3		0.022	−0.507 to 0.552
Beta-3 (rel), F7		−0.1499		Beta-3 (rel), F7		−0.007	−0.435 to 0.421
Alpha (rel), C4		−0.0694		Alpha (rel), C4		−0.253	−0.811 to 0.306
Alpha-2 (rel), C4		−0.0554		Alpha-2 (rel), C4		0.370	−0.396 to 1.135
Delta/Theta ratio, C4		0.1485		Delta/Theta ratio, C4		−0.358	−1.077 to 0.360
Theta (rel), F8		−0.1034		Theta (rel), F8		0.123	−0.627 to 0.873
High Beta (abs), T7		0.0548		High Beta (abs), T7		0.350	−0.240 to 0.941
High Beta (rel), T7		0.0673		High Beta (rel), T7		0.236	−0.314 to 0.785
Theta/Beta ratio, T8		−0.1768		Theta/Beta ratio, T8		0.317	−0.153 to 0.786
				(Constant)			−0.358 to 0.358

*rel, relative power; abs, absolute power*.

**Table 6:** Regressions on NSI cognitive.

**Elastic net regression**				**Linear regression**			
**Λ**	**α**	**DF**	**MSE**	***R* ^2^**	***F***	***p***	**MSE**
0.513	1	1	1.132	0.311	10.807	0.003	0.718
**Selected predictor**		**Beta at λ**		**Predictors**		**Beta**	**95% CI**
Theta/Alpha ratio, Pz		0.052		Theta/Alpha ratio, Pz		0.557^*^	0.207–0.907
				(Constant)			−0.343 to 0.343

* *p < 0.05*.

The remaining six outcome variables tested were not predicted by any of the 475 EEG variables entered in the LASSO regression models (Table 7). These outcomes included pain-related disability (CPG disability scale), somatic symptoms (NSI somatic subscale), vestibular symptoms (NSI vestibular subscale), sleep quality (MOS Sleep scale), depressive symptoms (PHQ-9), and migraine (MIDAS).

**Table 7:** Model fits for outcomes not predicted by EEG metrics.

**Outcome variable**	**Model fit**			
	**Λ**	**α**	**DF**	**MSE**
CPG disability	0.487	1	0	1.045
NSI somatic	0.433	1	0	1.035
NSI vestibular	0.423	1	0	1.149
MOS sleep	0.535	1	0	1.015
PHQ-9	0.458	1	0	1.075
MIDAS	0.595	1	0	0.984

## Discussion

In the current study, the delivery of LZT enabled us to investigate changes in EEG activity following an NFT protocol that is agnostic to specific EEG patterns or metrics. Given that our approach to LZT was unrestrained in terms of frequency bands and brain regions targeted during treatment, we sought to describe changes in EEG activity and how they relate to symptom improvements following LZT. The comparison of post-treatment to pre-treatment EEG activity revealed that patients most consistently increased frontal and central alpha activity. Our results also showed that changes in EEG activity differentially predict changes in specific symptoms. Due to the exploratory nature of this analysis and large number of comparisons, theoretical interpretations regarding these findings should be reserved for future confirmatory studies. However, these data generate important considerations regarding the development and refinement of NFT protocols for PPCS.

Our data suggest that when patients are free to implicitly modulate any oscillatory frequencies in any regions, individuals largely increase alpha activity brain-wide. Two-thirds of the EEG metrics that changed significantly following treatment were some measure of alpha activity—either relative alpha or a ratio including alpha—across the cortex. This suggests that modulation of alpha is implicitly favored over other frequencies in this specific NFT protocol. Clinicians using NFT protocols that target specific EEG patterns should consider that some frequencies in this population may be easier to modulate than others and accordingly adjust treatment parameters.

The predictive association observed between EEG activity and pain intensity has been previously reported by other authors (45). While our regression model was not statistically significant (likely due to limited power), it accounted for over 25% of the variance in the reduction in pain intensity, suggesting that these metrics contribute meaningfully to improving the subjective experience of pain in patients with PPCS. It is notable that of the 475 EEG variables entered into the model, three of the four selected as significant predictors of pain reduction were theta/alpha ratio, albeit in different regions. This pattern provides support for targeting theta/alpha ratio to treat pain in patients with PPCS. Currently, there is no evidence implicating a direct link between theta/alpha ratio and pain modulation; however, theta/alpha ratio is implicated in deep relaxation, hypnagogic states (46), and mood (47), which may mediate the relationship between NFT and pain modulation.

The predictive association between beta-related metrics and affective symptom improvements observed in our cohort is in agreement with extant evidence supporting the association of theta/beta ratio and beta power with emotion regulation (48), behavioral inhibition in emotional contexts (49), and affective processing (50). In consideration of this body of evidence, our results indicate that PPCS patients who report affective symptoms and/or emotion dysregulation may benefit from NFT that directly targets beta activity.

While it seems counterintuitive that a single EEG metric (theta/alpha ratio over the medial parietal region) accounted for 31% of the variance in cognitive symptom reduction, causal relationships between theta-targeted NFT and improved attention and working memory (51) as well as alpha-targeted NFT and improvements in spatial reasoning, executive function, and cognitive control, have been previously reported in non-clinical and non-PPCS cohorts. In conjunction with this previous evidence, our findings suggest that global cognitive improvement in this population may also be facilitated by targeting theta and alpha activity.

The six symptoms that were not predicted by EEG metrics tested in this analysis represent a variety of physical manifestations of mild TBI, including pain-related disability, somatic, vestibular, and depressive symptoms, poor sleep, and migraines. In contrast, we demonstrated that reduced pain intensity was predicted by changes in EEG activity following LZT. This dissociation suggests that NFT, while potentially effective for reducing pain, may not be sufficient to promote improvement for a broad range of physical symptoms for patients with PPCS; physical symptoms in this population may be treated more effectively using an integrative, multi-modal approach that includes NFT (52–54).

### Limitations and Future Directions

The feasibility study reported here used a single-group design; accordingly, changes in outcomes between pre- and post-treatment evaluations cannot be conclusively attributed to treatment effects. Future clinical trials with a control group are necessary to ascertain that the changes we observed are, in fact, due to LZT. Similarly, we cannot infer a causal relationship between changes in EEG activity and improvements in outcomes. Our regression-based analysis can only establish that changes in symptomatology are predicted by changes in EEG activity; experimental studies comparing outcomes following NFT that targets different EEG patterns may provide additional insight into causality.

Many of the linear regressions conducted in this study revealed high proportions of variance accounted for by the selected EEG metrics while the overall models did not achieve statistical significance (*p* < 0.05). The small sample size of this study, in combination with the reduction in power with increasing model complexity, likely impeded our ability to detect significant effects. Despite these statistical limitations, the proportions of variance explained by several of our models is large. Additionally, the distinction between statistical significance and clinical significance is of critical importance when evaluating the effects of NFT. Whereas, we highlighted the association between changes in EEG activity and reduced symptomatology, future research is warranted to examine the magnitude of change in EEG activity needed to produce clinically meaningful symptom reduction.

## Conclusions

The symptoms reported in PPCS are multifaceted and variable between patients; therefore, outcomes may be improved with greater treatment precision. Overall, we found evidence that changes to certain EEG metrics predict improvements in specific self-reported symptoms; taken together with previous findings that EEG activity in these specific frequency bands is abnormal in PPCS patients (55–57), our data corroborate existing evidence that these frequency bands may serve as critical targets for treating specific symptoms of PPCS. Specifically, we found evidence that changes in EEG activity following LZT—and NFT in general—are related to improvements in cognitive and affective symptoms, but less so for physical symptoms.

Our results describe changes in specific EEG metrics that predict reductions in specific self-reported symptoms, which indicates that unique symptom profiles of PPCS may be successfully targeted with individualized NFT protocols. This report ideally will promote further hypothesis testing of NFT for treating PPCS and promote the development and refinement of individualized, symptom-specific NFT protocols for patients with PPCS. Future research would benefit from stratifying patients in accordance with their symptom profiles or other phenotypical classifications to investigate the effects of LZT in distinct subtypes of patients and symptom clusters. In contrast, owing to its generalized and agnostic approach to targeting abnormal EEG activity, LZT has merit for treating PPCS in environments where generalized treatment is prioritized over individualized treatment, for example, if an NFT-trained clinician is unavailable or if specific symptoms cannot be dissociated or validly measured; however its superiority over more traditional and targeted forms of NFT remains to be demonstrated and warrants clinical trials (58).

## Data Availability Statement

The authors are ethically and legally restricted by the Madigan Army Medical Center Internal Review Board from sharing data because participants did not consent to this form of data sharing. However, we are able to provide data access to interested individuals pending Internal Review Board approval of individual data requests. Future interested individuals may contact the lead author, Jamie N. Hershaw, for queries and requests for data.

## Ethics Statement

The studies involving human participants were reviewed and approved by Madigan Army Medical Center IRB. The patients/participants provided their written informed consent to participate in this study. In the conduct of research where humans are the subjects, the investigators adhered to the policies regarding the protection of human subjects as prescribed by Code of Federal Regulations (CFR) Title 45, Volume 1, Part 46; Title 32, Chapter 1, Part 219; and Title 21, Chapter 1, Part 50 (Protection of Human Subjects).

## Author Contributions

CH-P contributed to the conception and design of the study and managed all study procedures and organized the database. JH performed the statistical analysis and wrote the manuscript. All authors contributed to manuscript revision and approved the submitted version.

## Funding

This research was funded by the Traumatic Brain Injury Center of Excellence (formerly Defense and Veterans Brain Injury Center) and the AMEDD Advanced Technology Initiative (AAMTI) through the Telemedicine and Advanced Technology Research Center (TATRC).

## Conflict of Interest

The authors declare that the research was conducted in the absence of any commercial or financial relationships that could be construed as a potential conflict of interest.

## Publisher's Note

All claims expressed in this article are solely those of the authors and do not necessarily represent those of their affiliated organizations, or those of the publisher, the editors and the reviewers. Any product that may be evaluated in this article, or claim that may be made by its manufacturer, is not guaranteed or endorsed by the publisher.
