# Supplementary material for: Changes in EEG Activity Following Live Z-Score Training Predict Changes in Persistent Post-concussive Symptoms: An Exploratory Analysis
Source: Front Neurol. 2022 Mar 21;13:714913. doi: 10.3389/fneur.2022.714913 (PMC8979790; doi:10.3389/fneur.2022.714913)
Supplement: Supplementary file 1 [file Table_1.DOCX]

| Supplementary Table A. Descriptive statistics for symptom change scores | | | | | | | |
| --- | --- | --- | --- | --- | --- | --- | --- |
|  | | | Range | | Quartiles | | |
|  | Mean | SD | Min | Max | Q1 | Q2 | Q3 |
| CPG Intensity | -3.975 | 12.627 | -33.670 | 23.000 | -9.670 | -3.165 | 3.670 |
| CPG Disability | -10.910 | 20.821 | -53.0 | 30.0 | -23.3 | -13.2 | 3.7 |
| NSI Somatic | -1.269 | 3.869 | -7.0 | 7.0 | -4.0 | -1.0 | 2.0 |
| NSI Affective | -1.115 | 4.718 | -10.0 | 9.0 | -4.0 | -1.0 | 1.0 |
| NSI Cognitive | -2.000 | 3.669 | -8.0 | 6.0 | -6.0 | -1.0 | 0.0 |
| NSI Vestibular | -0.308 | 1.876 | -3.0 | 5.0 | -2.0 | 0.0 | 1.0 |
| MIDAS | -0.5 | 60.862 | -80.0 | 225.0 | -31.0 | -2.0 | 1.0 |
| PCL-M | -2.038 | 12.574 | -33.0 | 29.0 | -7.0 | -2.5 | 3.0 |
| MOS Sleep | 0.115 | 5.879 | -11.0 | 16.0 | -4.0 | -1.0 | 3.0 |
| PHQ-9 | -2.000 | 5.107 | -14.0 | 9.0 | -5.0 | -2.0 | 0.0 |

| Supplementary Table B. Descriptive statistics for predictive EEG difference scores | | | | | | | |
| --- | --- | --- | --- | --- | --- | --- | --- |
|  | | | Range | | Quartiles | | |
|  | Mean | SD | Min | Max | Q1 | Q2 | Q3 |
| Delta (rel), FP1 | -3.828 | 22.213 | -58.291 | 46.865 | -14.413 | -1.205 | 5.000 |
| Beta-2 (rel), F3 | -0.005 | 0.021 | -0.061 | 0.027 | -0.019 | -0.003 | 0.011 |
| Theta/Alpha ratio, F3 | 0.052 | 0.303 | -0.622 | 0.901 | -0.152 | 0.005 | 0.182 |
| Beta (rel), C3 | -0.008 | 0.055 | -0.138 | 0.175 | -0.026 | -0.010 | 0.012 |
| High Beta (rel), C3 | 0.000 | 0.032 | -0.053 | 0.078 | -0.026 | -0.003 | 0.025 |
| Theta (rel), O1 | -0.024 | 0.063 | -0.137 | 0.123 | -0.076 | -0.013 | 0.000 |
| Theta/Alpha ratio, O1 | 0.034 | 0.191 | -0.249 | 0.568 | -0.069 | 0.015 | 0.128 |
| Beta-3 (rel), F7 | -0.003 | 0.010 | -0.029 | 0.013 | -0.008 | -0.003 | 0.005 |
| Alpha (rel), C4 | 0.010 | 0.019 | -0.035 | 0.051 | -0.004 | 0.010 | 0.025 |
| Alpha-2 (rel), C4 | 0.020 | 0.032 | -0.016 | 0.090 | -0.006 | 0.015 | 0.036 |
| Delta/Theta raio, C4 | 0.209 | 0.644 | -0.478 | 2.930 | -0.100 | 0.044 | 0.257 |
| Alpha/Beta ratio, P4 | -0.027 | 0.188 | -0.419 | 0.358 | -0.112 | -0.003 | 0.077 |
| Theta (rel), F8 | -0.031 | 0.089 | -0.216 | 0.230 | -0.093 | -0.043 | 0.021 |
| Theta/Alpha ratio, F8 | -0.049 | 0.251 | -0.726 | 0.403 | -0.201 | -0.076 | 0.127 |
| Alpha (rel), Cz | -0.002 | 0.033 | -0.072 | 0.076 | -0.024 | -0.004 | 0.021 |
| Beta-3 (rel), Pz | 0.002 | 0.010 | -0.021 | 0.022 | -0.002 | 0.000 | 0.012 |
| Theta/Alpha raio, Pz | 0.055 | 0.181 | -0.297 | 0.519 | -0.032 | 0.038 | 0.129 |
| Beta-2 (abs), T7 | -0.191 | 0.958 | -2.924 | 2.316 | -0.228 | -0.038 | 0.190 |
| High Beta (abs), T7 | -0.168 | 2.144 | -5.063 | 7.357 | -0.652 | -0.138 | 0.262 |
| High Beta (rel), T7 | -0.007 | 0.049 | -0.129 | 0.082 | -0.026 | -0.010 | 0.026 |
| Theta/Beta ratio, T8 | -0.011 | 0.078 | -0.117 | 0.153 | -0.072 | -0.036 | 0.051 |
